# RAL-1 signaling regulates lipid composition in *C. elegans*

**DOI:** 10.17912/micropub.biology.001054

**Published:** 2024-02-21

**Authors:** You Wu, Minjung Lee, A. Sena Mutlu, Meng Wang, David J. Reiner

**Affiliations:** 1 Department of Translational Medical Sciences, School of Medicine, Texas A&M University, Houston, TX; 2 Huffington Center on Aging, Baylor College of Medicine, Houston, Texas, United States; 3 Department of Molecular and Human Genetics, Baylor College of Medicine, Houston, Texas, United States; 4 Janelia Research Campus, Ashburn, Virginia, United States; 5 Institute of Biosciences and Technology, Texas A&M Health Science Center, Texas A&M University, Houston, TX

## Abstract

Signaling by the Ral small GTPase is poorly understood *in vivo* . *Caenorhabditis elegans* animals with constitutively activated RAL-1 or deficient for the inhibitory RalGAP, HGAP-1 /2, display pale intestines. Staining with Oil Red O detected decreased intestinal lipids in the *hgap-1* deletion mutant relative to the wild type. Constitutively activated RAL-1 decreased lipid detected by stimulated Raman scattering (SRS) microscopy, a label-free method of detecting lipid by laser excitation and detection. A signaling-deficient missense mutant for RAL-1 also displayed reduced lipid staining via SRS. We conclude that RAL-1 signaling regulates lipid homeostasis, biosynthesis or storage in live animals.

**Figure 1. RAL-1 regulates lipid homeostasis, biosynthesis and/or storage:**
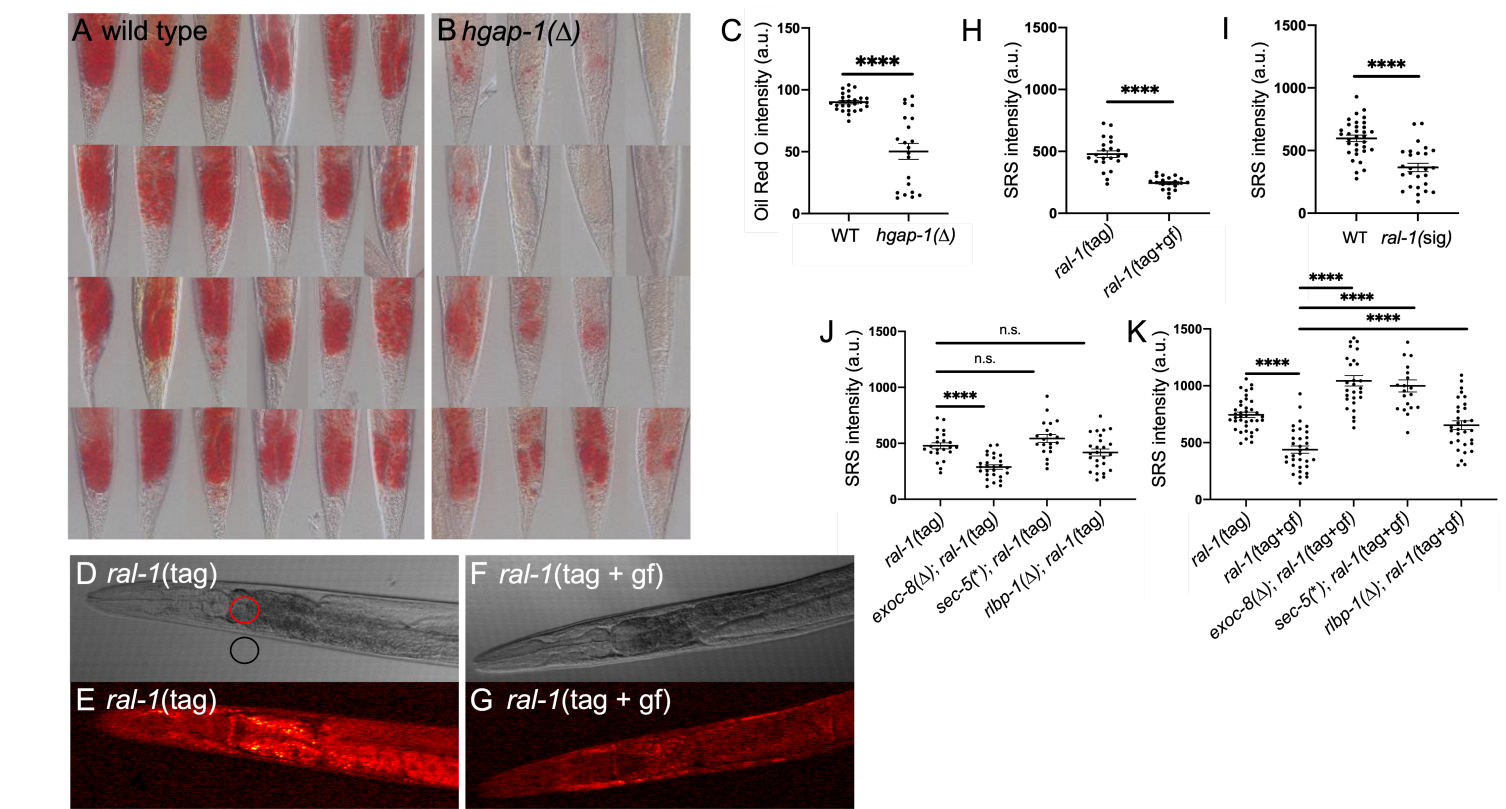
**A-C)** Using an Oil Red O staining protocol we found that lipid storage was decreased in **B)** *hgap-1 (* ∆ *)* relative to **(A)** wild-type animals. These data are quantitated in **(C)** as arbitrary units (a.u.) (P < 0.0001; posterior intestine showed). **D-H** ) Stimulated Raman Scattering (SRS) imaging of lipid levels revealed decreased lipid in animals with constitutively active RAL-1 . DIC ( **D, F** ) and SRS ( **E,G** ) imaging of animals with RAL-1 tagged at the N-terminal with mKate2::3xFlag. Wild-type ( **D, E** ) were compared to G26V constitutively activated RAL-1 ( **F, G** ). Pixel intensity was measured from the SRS images ( **E, G** ) in the areas illustrated with red and black circles of 35 μm ( **D** ). Background (black circle) was subtracted from the anterior intestine (red circle) in the SRS image to yield a value for lipid content. These data for various experiments were graphed as normalized SRS intensity ( **H-K** ). **H** ) Comparison of tagged wild-type vs. G26V constitutively activated RAL-1 animals. **I** ) Comparison of wild-type vs. *ral-1 (* sig *)* ( *ral-1 ( gk628801 [R139H])* signaling deficient RAL-1 (no tag for either). **J** ) Comparison of tagged RAL-1 single mutant or double mutants with mutations in *exoc-8* , *sec-5* , or *rlbp-1* . *exoc-8 (* Δ *)* is the *ok2523* deletion, *rlbp-1 (* Δ *)* is the *tm3665* deletion, and *sec-5 (* * *)* causes a premature stop at codon 369 of 884 residues in SEC-5. **K** ) Comparison of tagged wild-type or G26V constitutively active RAL-1 single mutant or double mutants with mutations in *exoc-8* , *sec-5* , or *rlbp-1* . Data within each panel were scored concurrently but data between panel scored on different days. (Note difference in baseline of tagged RAL-1 between panels **J** and **K** . Data in **C** , **H** , **I** , **J** and **K** were subjected to the T-test.) Error bars represent SEM. *<0.05, **<0.01, ***<0.001, ****<0.0001, n.s. = not significant.

## Description

The Ras small GTPase is the most mutated human oncoprotein: 19% of tumors harbor activating mutations in Ras (Prior, Hood, & Hartley, 2020) . Oncogenic Ras utilizes three main direct binding partners, called effectors, that propagate downstream signaling. The Raf>MEK>ERK MAP Kinase pathway and PI3K>PDK>AKT pathway are among the best studied and pharmacologically targeted signaling cascades in all of biology (Cox et al., 2014) . In contrast, Ras activation of RalGEF signaling through Ral (RalGEF>Ral) is neglected and poorly understood, despite playing a critical role in Ras-driven tumorigenesis (Apken & Oeckinghaus, 2021) . The inhibitory GAP for Ral is also implicated as a tumor suppressor, suggesting that Ral can drive tumorigenesis in the absence of activating mutations in Ras (Beel et al., 2020; Oeckinghaus et al., 2014; Yoshimachi et al., 2021) .

Ral ( Ra s l ike) is a small GTPase in the Ras family. Ras itself is the founding member of the Ras superfamily of small GTPases. GTP-bound Ral (Ral·GTP) is in the active state and engaging downstream effectors, while GDP-bound Ral (Ral·GDP) is in the inactive state. RalGEF ( g uanine nucleotide e xchange f actor) is bound by activated Ras to stimulate nucleotide disassociation of Ral·GDP, upon which free cytosolic GTP spontaneously loads to form Ral·GTP. RalGAP ( G TPase a ctivating p rotein) stimulates the poor intrinsic GTPase activity of Ral to hydrolyze GTP to GDP to yield Ral·GDP, hence inactivating Ral. These general mechanisms for regulating Ral are conserved among the Ras superfamily, including Ras itself and related families Rho, Rab, Arf and Ran (Reiner & Lundquist, 2018; Wennerberg, Rossman, & Der, 2005) .

In *C. elegans* , signaling via LET-60 /Ras> RGL-1 /RalGEF> RAL-1 /Ral via a downstream GCK-2 /CNH-MAP4 Kinase> PMK-1 /p38 MAP kinase cascade promotes 2˚ vulval precursor cell fate in support of LIN-12 /Notch (Shin et al., 2019; Shin et al., 2018; Zand, Reiner, & Der, 2011) . RGL-1 /RalGEF> RAL-1 /Ral also contributes broadly to cell migration events in the animal (Mardick et al., 2021) .

The mammalian RalGAP is a heterodimeric protein (two alpha- and one beta-subunit-encoding genes) (Chen et al., 2011) . *C. elegans* encodes one alpha subunit, HGAP-1 (for h eterodimeric GAP ), and one beta subunit, HGAP-2 . Loss of HGAP1/2 function results in decreased lifespan while loss of RAL-1 /Ral extends lifespan (Martin et al., 2014) .

By visual inspection, we previously observed that hgap-1 ( gk101481 [W1142*]) and hgap-2 ( gk578143 [Q802*]) nonsense mutant animals exhibited pale intestines in late L4 and adult. The *hgap-1 ( tm6435 )* deletion mutation conferred the same pale intestine phenotype. The *ral-1 ( re160* gf *[mKate2::3xFlag:: RAL-1 (G26V)])* animal with constitutively activated RAL-1 /Ral displayed a similar phenotype. The intestines of these mutants on NG plates appeared to occlude light less well than wild-type animals, suggesting defects in either feeding, metabolism or fat storage (Lakowski & Hekimi, 1998) . Yet pumping for all these strains appeared normal. We hypothesized that excessive activation of RAL-1 causes altered metabolism or storage of lipids, which comprise the main light-occluding property of the *C. elegans* intestine (O'Rourke et al., 2009).

To test this hypothesis, we fixed and stained *hgap-1 ( tm6435 )* putative null mutant animals with Oil Red O, a dye that binds lipid compartments (Wahlby et al., 2014). We observed significant decrease in lipid staining in *hgap-1* mutant vs. wild-type animals ( **Fig. 1A -C** ). We subsequently analyzed constitutively activated *ral-1 ( re160* gf *[mKate2::3xFlag:: RAL-1 (G26V)])* vs. wild-type *ral-1 ( re218 [mKate2::3xFlag:: RAL-1 (+)])* animals. (The tag with red fluorescent protein mKate plus 3xFlag did not alter signaling properties of RAL-1 (Shin et al., 2018) ). We measured lipid storage with stimulated Raman scattering (SRS), a laser-based, label-free assay for lipid composition in animals (Mutlu et al., 2021; Wang et al., 2011) . SRS is unaffected by the presence of mKate2 red fluorescent protein in the animal because the laser used in SRS does not excite mKate in the necessary wavelength. Constitutively activated RAL-1 conferred decreased lipid composition relative to the wild type ( **Fig. 1D -H** ).

We have also characterized the *ral-1 ( gk628801 [R139H])* mutant, which abolishes 2˚ VPC-promoting signal and compromises cell migration events, but which is otherwise superficially wild type (Mardick et al., 2021; Shin et al., 2019; Shin et al., 2018) . These signaling deficient animals also display decreased fat content by SRS imaging ( **Fig. 1I** ). However, we could not perceive a pale intestine phenotype associated with signaling deficient RAL-1 , which suggests that the mechanism of decreased lipid detectable by SRS is distinct from that observed from increased RAL-1 signaling.

Like Ras, mammalian Ral proteins have three principal oncogenic effectors/binding partners: Sec5 and Exo84 of the exocyst complex and RalBP1. These proteins regulate exocytosis and trafficking activities in the cell but also mediate downstream Ral signaling via unknown mechanisms (Apken & Oeckinghaus, 2021; Gentry et al., 2014; Kashatus, 2013) . Applying the same SRS method as above, we observed that the *exoc-8 ( ok2523 )* deletion mutation decreased fat content but deletion mutant *rlbp-1 ( tm3665 )* and nonsense mutant from heterozygous mother, *sec-5 ( pk2357 )* , caused no defect ( **Fig. 1J** ). In a separate experiment, mutation of *exoc-8* and *sec-5* reversed the decreased fat content of *ral-1 ( re160* gf *)* animals, resulting in increased fat content ( **Fig. 1K** ). Deletion of *rlbp-1* also reversed the decreased fat content of the constitutively activated RAL-1 .

We conclude that increase of Ral activation, either through gain of RAL-1 function or loss of inhibitory HGAP, reduces detectable fat content as detected by Oil Red O or SRS. Reduction of RAL-1 signaling activity as assayed in our studies also resulted in decreased lipids, but likely via a distinct mechanism. Though perhaps paradoxical, these observations could reflect differences in mobilization of detectable lipids or metabolism to different molecular species.

The effects of deletion of putative effectors of RAL-1 lead to uninterpretable results. Yet perhaps this is not surprising: these proteins perform an array of complex cellular functions beyond signal transduction. Exo84 and Sec5 are components of the heterooctameric exocyst complex, which performs cell-essential functions in direct exocytosis via the Golgi (Pereira et al., 2023) . The exocyst is evolutionarily conserved from yeast to mammals, but unlike metazoans, yeasts do not encode Ral orthologs. In addition to being bound by Ral·GTP, RalBP1 ( Ral b inding p rotein 1) functions as a GAP to inhibit Rac and Cdc42 of the Rho family of small GTPases, primarily known for regulating cytoskeletal dynamics. RalBP1 also functions as a non-ABC ATP-dependent transporter with ATP-binding sites, regulates mitochondrial fission/fusion, and associates with EH domain-containing proteins REPS1 and POB1, which function in receptor-mediated endocytosis. (Cornish, Owen, & Mott, 2021) . Consequently, all three putative effectors of RAL-1 could be expected to exert complex influence on lipid biosynthesis and/or storage in the animal. Better understanding of these regulatory inputs into storage and metabolism of lipids will require selective missense mutations that uncouple specific functions, partnered with more complex analysis of lipid metabolism.

## Methods

Animals were cultured at 20˚C on NGM plates spotted with OP50 *E. coli* bacteria. All strains are derived from the N2 Bristol wild-type background.

For staining via Oil Red O (ORO), animals were fixed with isopropanol and stained with ORO dye as described (O'Rourke et al., 2009; Wahlby et al., 2014). ORO data were acquired using a Nikon eclipse Ni microscope via epifluorescence or DIC/Nomarski imaging with a Nikon DS-Fi2 color camera. Images were processed using NIS-Elements Advanced software Research, Version 4.40. ORO intensity measurement was performed using Fiji Image J software version 2.1.0/1.53C (NIH). Original color image documents were split into red, green, blue channels (Image → color → split channels). Intensity of red channel was obtained by subtracting blue and green pixel intensity (creating blue + green channel: Process → image calculator. image1: green/ image2: blue/ Operation: average; creating red-only channel: Process → image calculator. image1: red/ image2: result of green + blue channels/Operation: subtract). Average intensity was measured from a circle of 150 pixels in a diameter in the posterior intestine of each animal.

For lipid detection via Stimulated Raman Scattering (SRS), animals were anesthetized with 10 mM tetramisole, mounted on 2% agar pads on glass slides, and subjected to laser stimulation and confocal microscopy image capture as described (Mutlu et al., 2021; Wang et al., 2011) . Briefly, an Olympus IX81 inverted laser-scanning confocal microscope optimized for near infrared signal detected signal generated by temporally overlapping Pump and Stokes laser beams and optimized for lipids contained in lipid droplets (Mutlu et al., 2020) . Images were processed using Olympus Fluoview 1000 software.

## Reagents

**Table:** 

**Strain #**	**Genotype**	**Source**
DV3238	*ral-1 ( re160* gf *[mKate2::3xFlag::RAL-1(G26V)])* III	Shin 2018
DV3402	*ral-1 ( re218 [mKate2::3xFlag::RAL-1(+)])* III	Shin 2018
DV2942	*ral-1 ( gk628801 [R139H])* III 6x outcrossed	Shin 2018
DV3297	*sec-5 ( pk2357 )* / *mIn1 [ dpy-10 ( e128 ) mIs14 (myo-2p>GFP)]* II	Shin 2018
DV2690	*rlbp-1 ( tm3665 )* I 5x outcrossed	Shin 2018
DV3202	*exoc-8 ( ok2523 )* I 3x outcrossed	Shin 2018
DV2902	*hgap-1 ( tm6435 )* I 3x outcrossed	This study
DV2824	hgap-1 ( gk101481 [W1142*]) I 3x outcrossed	This study
DV2815	hgap-2 ( gk578143 [Q802*]) II 2x outcrossed	This study
DV3853	*exoc-8 ( ok2523 )* I; *ral-1 ( re218 [mKate2::3xFlag::RAL-1(+)])* III	This study
DV3854	*sec-5 ( pk2357 )* / *mIn1 [ dpy-10 ( e128 ) mIs14 (myo-2p>GFP)]* II; *ral-1 ( re218 [mKate2::3xFlag::RAL-1(+)])* III	This study
DV3866	*rlbp-1 ( tm3665 )* I; *ral-1 ( re218 [mKate2::3xFlag::RAL-1(+)])* III	This study
DV3300	*exoc-8 ( ok2523 )* I; *ral-1 ( re160* gf *[mKate2::3xFlag::RAL-1(G26V)])* III	This study
DV3818	*sec-5 ( pk2357 )* / *mIn1 [ dpy-10 ( e128 ) mIs14 (myo-2p>GFP)]* II; *ral-1 ( re160* gf *[mKate2::3xFlag::RAL-1(G26V)])* III	This study
DV3817	*rlbp-1 ( tm3665 )* I; *ral-1 ( re160* gf *[mKate2::3xFlag::RAL-1(G26V)])* III	This study
